# Development of a deep learning-based error detection system without error dose maps in the patient-specific quality assurance of volumetric modulated arc therapy

**DOI:** 10.1093/jrr/rrad028

**Published:** 2023-05-12

**Authors:** Yuto Kimura, Noriyuki Kadoya, Yohei Oku, Keiichi Jingu

**Affiliations:** Department of Radiation Oncology, Tohoku University Graduate School of Medicine, 1-1 Seiryo-machi, Aoba-ku, Sendai 980-8574, Japan; Radiation Oncology Center, Ofuna Chuo Hospital, 6-2-24 Ofuna, Kamakura, 247-0056, Japan; Department of Radiation Oncology, Tohoku University Graduate School of Medicine, 1-1 Seiryo-machi, Aoba-ku, Sendai 980-8574, Japan; Radiation Oncology Center, Ofuna Chuo Hospital, 6-2-24 Ofuna, Kamakura, 247-0056, Japan; Department of Radiation Oncology, Tohoku University Graduate School of Medicine, 1-1 Seiryo-machi, Aoba-ku, Sendai 980-8574, Japan

**Keywords:** radiotherapy, patient-specific QA, deep learning, VAE

## Abstract

To detect errors in patient-specific quality assurance (QA) for volumetric modulated arc therapy (VMAT), we proposed an error detection method based on dose distribution analysis using unsupervised deep learning approach and analyzed 161 prostate VMAT beams measured with a cylindrical detector. For performing error simulation, in addition to error-free dose distribution, dose distributions containing nine types of error, including multileaf collimator (MLC) positional errors, gantry rotation errors, radiation output errors and phantom setup errors, were generated. Only error-free data were employed for the model training, and error-free and error data were employed for the tests. As a deep learning model, the variational autoencoder (VAE) was adopted. The anomaly of test data was quantified by calculating Mahalanobis distance based on the feature vectors acquired from a trained encoder. Based on this anomaly, test data were classified as ‘error-free’ or ‘any-error.’ For comparison with conventional approaches, gamma (γ)-analysis was performed, and supervised learning convolutional neural network (S-CNN) was constructed. Receiver operating characteristic curves were obtained to evaluate their performance with the area under the curve (AUC). For all error types, except systematic MLC positional and radiation output errors, the performance of the methods was in the order of S-CNN ˃ VAE-based ˃ γ-analysis (only S-CNN required error data for model training). For example, in random MLC positional error simulation, the AUC of our method, S-CNN and γ-analysis were 0.699, 0.921 and 0.669, respectively. Our results showed that the VAE-based method has the potential to detect errors in patient-specific VMAT QA.

## INTRODUCTION

Patient-specific quality assurance (QA) is performed to confirm if a treatment plan is clinically acceptable before a patient undergoes intensity-modulated radiation therapy (IMRT) [1, 2]. The reproducibility of the treatment plan generated by a treatment planning system (TPS) is verified by comparing the output from a linear accelerator, which is acquired using a measurement device, with the dose distribution predicted using TPS. In patient-specific IMRT QA, gamma (γ)-analysis is generally employed [3]. However, several studies have reported that γ-analysis may be less sensitive to errors in the dose distribution [4–9]. Yan *et al*. [7] simulated MLC positional errors in the static beam IMRT QA. Their results showed that the average γ-pass rate for plans with random multileaf collimator (MLC) positional errors exceeded 90%, with a small difference from the pass rate for error-free plans. Therefore, if a mechanical error occurs, it may be undetected by the γ-analysis method in patient-specific QA.

Recently, in the IMRT dosimetry QA, machine learning-based approaches have been reported for dose-distribution analysis as methods that can improve error sensitivity [10–17]. Wootton *et al*. [10] investigated the performance of an MLC positional error detection method combining radiomics [18–22] and machine learning approaches in the static beam IMRT QA by performing simulations with the MLC error-introduced plan. They generated the γ-map from the measured and predicted fluence maps in an electronic portal imaging device (EPID) and analyzed the map using radiomics. Potter *et al*. [14] reported an error detection model using a deep learning approach for patient-specific static beam IMRT QA using a 2D diode array. Their model showed the possibility of detecting not only the MLC positional error but also the errors of MLC transmission, monitor unit, effective source size and alignment of a measuring device.

Although the previous models are sensitive to errors, they have a drawback due to the adaptation of supervised learning for model training. The drawback is that the models cannot handle unknown error types unexpected in advance. When applying a supervised learning approach, the dose distributions containing the errors to be detected and labels for the errors are required for model training. In addition, it is necessary to determine the structure of the machine learning model in advance according to the number of error types to be detected. Recent machine learning-based models have been extended to detect some types of mechanical errors. However, in clinical practice, many error patterns, including those based on mechanical and plan-specific factors, can occur. Therefore, it is difficult to predict in advance all possible error patterns and prepare a large amount of data for all error patterns for training. Thus, these approaches cannot replace γ-analysis in patient-specific QA but can only supplement it. To the best of our knowledge, there are no reports of machine learning approaches to address this issue.

To address these problems, we proposed an error detection method utilizing an unsupervised learning-based neural network model that does not require the error dose data during the model training process. To implement this method, we adopted a variational autoencoder (VAE) [23] as a machine learning model. This study aims to develop a VAE-based error detection method and evaluate its performance by simulating error-introduced plans in patient-specific volumetric modulated arc therapy (VMAT) QA. To clarify the performance as an error classifier, we performed the receiver operating characteristic (ROC) analysis and compared the performance of the proposed VAE-based, conventional supervised learning convolutional neural network (S-CNN) and conventional γ-analysis methods.

## MATERIAL AND METHODS

### Treatment plans and the dose-distribution measurement

A total of 104 prostate VMAT plans with 161 beams from patients who were treated at our hospital were employed retrospectively. For the field design, one or two full arcs were used, and a beam energy of 10 MV was applied. Irradiation was performed with a Clinac iX linear accelerator with a Millennium 120 MLC (Varian Medical Systems, Palo Alto, CA). The clinical target volume was defined as either prostate only or prostate plus seminal vesicles, and the prescribed dose was 1.8–7.25 Gy per single fraction.

All plans were transferred to the computed tomography set of a cylindrical phantom, and the dose distributions were calculated using Eclipse 15.6 TPS (Varian Medical Systems) with the Acuros XB algorithm. The grid size was set to 2.5 × 2.5 × 2.5 mm [3]. Next, the dose-distribution output from the linear accelerator were measured using the Delta4 system (ScandiDos, Uppsala, Sweden). The beams were delivered to the Delta4 phantom (a cylindrical phantom), which contained 1069 diodes in two orthogonal planes, inclined at 50° and 40° clockwise and counterclockwise from the vertical axis, respectively [24–27]. The measured dose distributions were corrected with the output correction factor obtained on each measurement day based on a 10 × 10-cm [2] square field. To simulate the situation of minimal phantom setup errors, we applied the optimized phantom position function. This function, which is included in the analysis software dedicated to Delta4, was corrected for phantom setup errors by calculating the amount of phantom shift that increased the γ-pass rate. In this study, the criterion of dose difference (DD) and distance to agreement (DTA) for the calculation of the γ-pass rate was set to 3%/1 mm (DD/DTA) to increase the sensitivity to positional errors.

### Creation of the plans with introduced errors

In addition to the clinically employed plan (denoted as error-free plan), plans with some types of errors were created to perform a simulation to evaluate the performance of our method. The errors were assumed to be the MLC positional, gantry rotation, radiation output and phantom setup errors.

For the MLC error simulation, two types of plans with introduced errors, either systematic or random, were generated. The method of error modeling was reported in our previous study [13]. Briefly, for the plans with systematic errors, the leaf positions were offset by 2 mm in a single direction, whereas, for those with random errors, the leaf positions were offset randomly according to a Gaussian distribution of a 2-mm width (1 sigma). The dose distributions in the cylindrical phantom were calculated for the two types of MLC error plans using Eclipse.

The dose distributions of the other errors were generated by editing the doses in the cylindrical phantom of the error-free plans. The gantry rotation errors were reproduced by rotating the error-free dose, clockwise and counterclockwise, by 2°. The dose distributions with the introduced radiation output errors were created by renormalizing the error-free dose to $\pm$3%. As for the phantom setup error, three patterns of errors were reproduced by resampling the error-free dose distribution so that it was shifted by 1 mm in each of the lateral, longitudinal and vertical directions.

### Generating the datasets for VAE

As an input to the VAE model, the DD maps were generated from the calculated and measured dose distributions. As the calculated doses, both the error-free and error-containing doses were used. First, both the calculated and measured dose distributions were normalized by an isocenter dose measured using Delta4 to eliminate the effects of the difference in prescription dose between the plans. Next, the point doses at the point where the diode of the Delta4 phantom was built-in were extracted from the calculated dose distributions. The DD maps were obtained from the measured dose distributions in Delta4, and the point doses were extracted from the calculated dose distributions using TPS. The Delta4 had diodes spaced 5 or 10 mm apart, which were centered at the isocenter of the diode. Therefore, to generate a map that matches the minimum resolution of 5 mm, interpolation processing was applied only to areas that were not covered by the diodes’ built-in positions alone. Since the distance between measurement points in Delta4 is wider than that of EPID and other systems, the application of excessive interpolation processing may result in inaccurate information being reflected in the map, especially in areas with steep dose gradients. Therefore, interpolation processing for generating higher resolution maps was not performed. From this map, a 27 × 27 matrix size map with a central 13 × 13 cm [2] area extracted was used as the input data to VAE. Finally, the threshold processing of all maps was performed based on the upper and lower limits, i.e. +0.2 and −0.2. If test data containing a DD larger than the DD input in the training process are input to the model, it may affect the output of the model, so the threshold processing was performed by the same method as in the previous study [13].

Among the 161 beams, 125 were randomly allocated to the training process and 36 were allocated to the testing process. Because only the input maps generated from the error-free dose data were required for the model training process, we prepared 200 input maps for 100 beams as training data and 50 input maps for 25 beams as validation data. Further, the input maps of the training data were expanded 4-fold by flipping the original image horizontally, upside down and both. For the model testing, nine types of errors were assumed, the error-free patterns were also added and the dose distributions of 10 patterns per beam were generated. Therefore, 720 input maps were generated from the 36 measured dose distributions and the 360 calculated dose distributions for testing.

### Construction of the VAE model and training

**Fig. 1:**
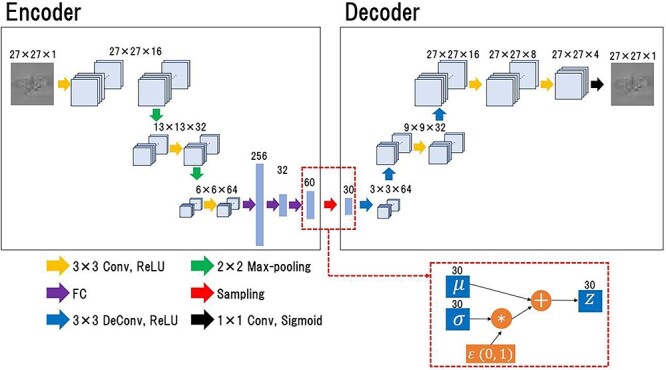
The architecture of the VAE model. The encoder receives a DD map and outputs the parameters, *μ* and *σ*, which determine the distribution of the latent variable. The mean parameter, *μ*, and the variance parameter, *σ*, are employed to create a distribution for sampling the latent variables. The latent variable, *z*, is passed to the decoder via the sampling step, and the decoder reproduces the input image from the latent variables.

A VAE model was constructed to quantify the anomaly of the DD map. Figure 1 shows the VAE model architecture. The model comprised two parts: the encoder, which outputs the compressed representations of the input image; and the decoder, which reconstructs the original input image from the latent variables by scaling up with the deconvolution layers. Unlike a general autoencoder, an encoder of VAE did not directly output the latent variables to be passed to the decoder but output the parameters that determine the distribution of the latent variables. The output parameters from the encoder are two vectors, $\mu$ and $\sigma$, representing the mean and variance, respectively, of the latent state distribution, and the two vectors were employed to generate a distribution to sample the latent variables. The latent variables were calculated as follows [23]:


(1) \begin{equation*} z=\mu +\varepsilon \cdotp \sigma, \end{equation*}

where $z$ is the latent variable to be passed to the decoder and $\varepsilon$ is a random number between 0 and 1.

For training the model, the loss function, also called the evidence lower bound loss, was calculated as follows [23]:


(2) \begin{equation*} \mathrm{ELBO}\ \mathrm{loss}=\mathrm{Reconstruction}\ \mathrm{loss}+\mathrm{KL}\ \mathrm{loss}, \end{equation*}

where the reconstruction loss is the mean-squared error between the input and reconstructed images, and the second term is the Kullback–Leibler (KL) divergence, which is a measure of the difference between the two probability distributions. These two distributions indicate the latent state distribution determined by the parameter output from the encoder and a Gaussian distribution of mean 0 and standard deviation 1. Here, the KL loss is a closed-form solution, under Gaussian assumptions. For the *n*-dimensional latent variables, the KL loss was calculated as follows:


(3) \begin{equation*} \mathrm{KL}\ \mathrm{loss}=-\frac{1}{2}\sum_{i=1}^n\left(1+\log{\sigma}_i-{\mu}_i^2-{\sigma}_i^2\right). \end{equation*}

By adding the KL loss to the loss function, the parameters of the latent variable, that was obtained by the reconstruction loss, were limited to around the center of the latent space and featured a continuous distribution.

The VAE model was constructed and trained using MATLAB 2020a (Math Works, Natick, MA, USA). The model training was performed with only the error-free dose maps. To ensure good performance, the number of epochs was set to 200, and the minibatch size was set to 64. The adaptive moment estimation was the optimization algorithm for model training.

### Quantification of the anomaly in the dose maps for VAE-based approach

To classify the test data into the error-free or ‘any-error’ class, a method that quantifies the dose map anomaly based on error-free data was implemented. After the model training process, training and test data were input into the model, and the feature vectors were acquired from the encoder. Based on the feature vectors $\mu$, Mahalanobis distance (MD) [28] was calculated as the degree of an anomaly in the dose maps. MD facilitated the calculation of the distance between the data by considering the correlation of the data through the inverse of the variance–covariance matrix ($\Sigma$) of the target dataset. Therefore, MD quantified the degree of abnormality of data based on the standard data. Here, $\mu$ obtained by encoding the training dataset, that comprised error-free maps, was used as the standard parameter for MD calculation. Then, based on the standard data, the anomaly degree in the dose map of the test dataset was quantified by calculating the MD using $\mu$ obtained by encoding the test dataset. In the case of a dataset comprising $n$ objects, MD for each object ${x}_i$ could be calculated as follows:


(4) \begin{equation*} {\mathrm{MD}}_i=\sqrt{\left({x}_i-\overline{x}\right){\Sigma}^{-1}{\left({x}_i-\overline{x}\right)}^T}\ \mathrm{for}\ i=1\ \mathrm{to}\ n, \end{equation*}

where $\Sigma$ is the variance–covariance matrix. MD was calculated based on the all-dimensional component of $\mu$. To quantify the anomaly in the test dose maps based on the error-free dose maps, which were employed as training data, $\Sigma$ and the mean value, $\overline{x,}$ which were obtained from $\mu$ acquired by encoding the training data, were adopted for the calculation.

Because Delta4 possesses two measurement planes, two input maps were generated from a pair of measured and calculated dose distributions. Therefore, the mean MD was employed to classify all the 360 cases into two classes based on the analysis results of the two maps. If the calculated MD was lower than the threshold distance, it would be classified as ‘error-free,’ and if it exceeded the threshold distance, it would be classified as ‘any-error.’ The two-class classification was performed based on MD, and the ROC curves were generated. More details on ROC analysis are provided below.

### Comparison with S-CNN

To compare the performance of our method, we constructed the conventional CNN model using supervised learning. It was a simple model for classifying the input map into the ‘error-free’ or ‘any-error’ class. The architecture consisted of three convolution blocks followed by fully connected layers and a softmax layer. In addition, each convolution block comprised a convolution layer of kernel size 3 × 3, a batch normalization layer, a ReLU layer and a 2 × 2 max-pooling layer. The number of filters for convolution was set to 16, 32 and 64 to ensure that the upstream block was close to the encoder in VAE, and the number of nodes in the fully connected layer was set to 256, 32 and 2 from the upstream. There were two dropout layers between the three fully connected layers, and their dropout rates were set to 0.2 and 0.5, respectively, from the upstream to ensure good performance. The model outputs error-free and any-error probabilities from a single input map.

The model was trained with input maps acquired from clinical plans labeled as ‘error-free’ and nine types of error input maps labeled as ‘any-error.’ Similar to the VAE model training and testing process, of the 161 beams, 100 were allocated as the training data, 25 were allocated as the validation data for model training process and 36 were allocated to the testing process. Because one dose distribution generated two input maps, 200, 50 and 72 error-free maps were generated and 1800, 450 and 648 error maps were generated for training, validation and testing datasets, respectively. To equalize the number of error-free maps and any-error maps for model training, 200 error-free maps were expanded 9-fold through image flipping and rescaling. After the model training process, test datasets were analyzed by the trained CNN model. The error probabilities output from the two input maps of the pair were averaged, and 360 cases of the test dataset were classified as ‘error-free’ or ‘any-error’ based on the threshold of error probability. To evaluate the performance of the S-CNN, the ROC analysis was performed. More details on ROC analysis are provided below.

### Comparison with γ-analysis

To compare the performance of the proposed method, γ-analysis was performed using test sets. For performing analysis, in-house γ-analysis software was used. The γ-pass rate for each test set was calculated employing the two planar dose distributions with a built-in detector. The regions with ˂10% of the maximum dose area were excluded from this analysis, and the criteria of DD and DTA were set to the global 3%/2 and 2%/1 mm (DD/DTA). The criterion for the global 3%/2 mm was adopted as the generally employed clinical one [29]. Further, the criterion for 2%/1 mm was a strict one that is not generally employed clinically but was adopted as an example with an extremely high sensitivity to errors.

If the γ-pass rate exceeded the threshold value, it would be classified as ‘error-free’; otherwise, it would be classified as ‘any-free.’ The two-class classification was performed based on MD, and the ROC curves were generated. More details on ROC analysis are provided below.

### ROC analysis

ROC analysis was performed to evaluate the error detection performance of the proposed VAE-based, S-CNN and γ-analysis methods. Error classification in the ROC analysis process was performed based on the indicators of the anomaly of the input data calculated by analyzing the dose map of the test set with the three methods. The indicator of the anomaly of the dose map was MD, error presence probability and γ-pass rate for the proposed VAE-based, S-CNN and γ-analysis methods, respectively. The ROC curves were generated for each of the nine error type cases by plotting sensitivity versus 1$-$specificity. Sensitivity and specificity for generating ROC curves were acquired by performing two-class classification with the error-free and ‘any-error’ cases as the negative and positive classes, respectively. To evaluate the performance of the methods, the area under the curve (AUC) was calculated.

Further, by identifying the point closest to (0, 1) in the ROC space, the ideal thresholds for classification were determined for each method and each error pattern. Accuracy was calculated based on the results of the two-class classification performed at ideal thresholds.

## RESULTS

**Fig. 2:**
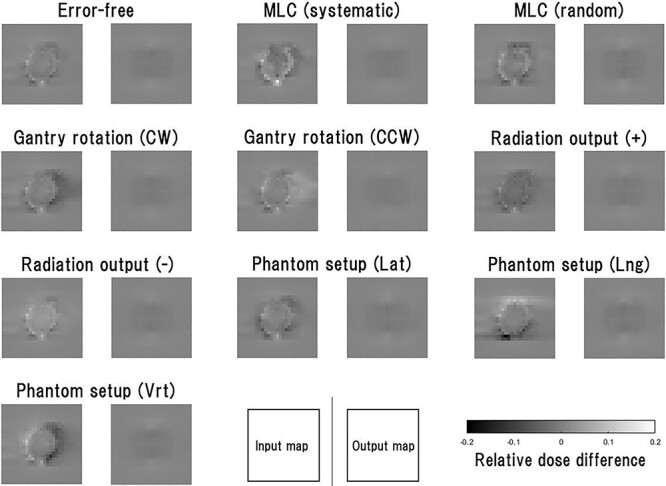
Examples of the input maps to the VAE model and the output maps from it for the test data. The input map represents the differences between the normalized measured and calculated maps, with threshold processing based on the upper and lower limits, i.e. +0.2 and −0.2. The test data comprise the maps in which the error-free and nine types of error cases were assumed. All the input maps were generated from the same single-beam measurement data, and the 10 types of dose distributions were generated using TPS and in-house software.

Examples of the input maps of the test data and the reproduced maps are shown in Fig. 2. In the error-free case, the input map of training data was an almost plain image, so the reproduced map was also a plain image. In any of the error cases, the input maps revealed unique features that reflected each error, although those features were excluded from the reproduced images.

**Fig. 3:**
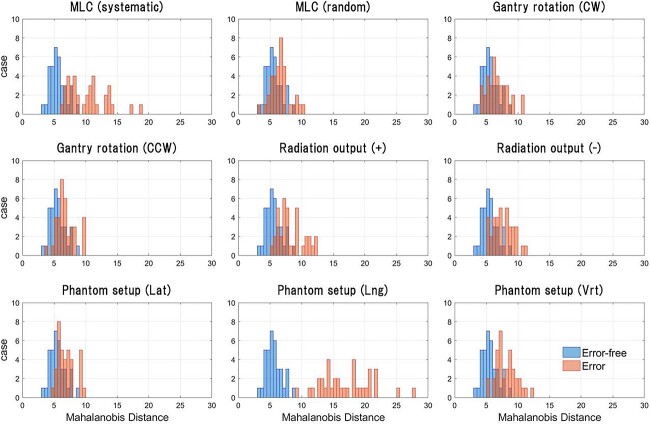
Result of anomaly quantification in the dose maps of the test dataset. Anomaly in the dose maps was quantified by calculating MD using *μ*, which was obtained by encoding the training and test data. The test data contained 360 cases, comprising 10 types of maps, including 1 error-free case and 9 ‘any-error’ cases. All types of test data contained 36 cases each. Based on the quantified anomaly, a two-class classification was performed, and ROC curves were generated for each type of error.

Figure 3 shows the MD obtained from the test dataset, where the MD indicates the anomaly degree of the dose map. The test dataset comprised 10 types of maps, including 1 error-free case and 9 ‘any-error’ cases, with 36 data per type.

**Fig. 4:**
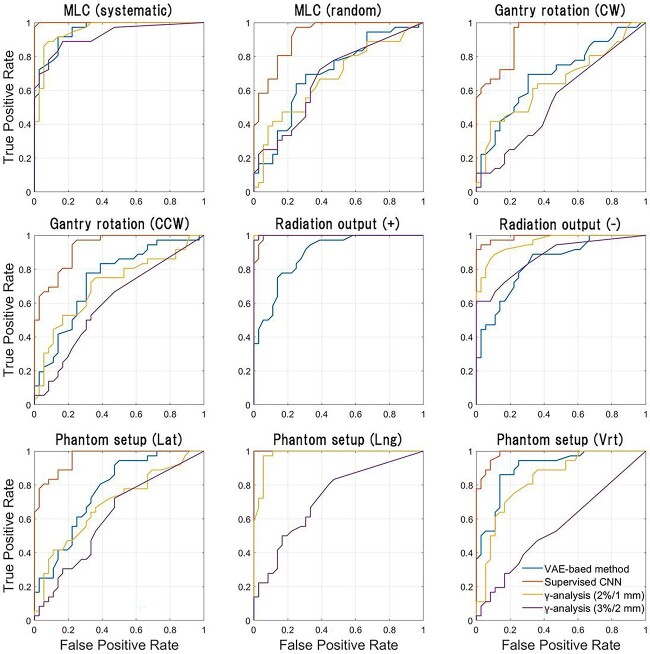
ROC curves for our method, S-CNN and conventional γ-analysis. To generate ROC curves while changing the threshold for classification, a two-class classification was performed for each type of error, with the error class as the positive class and the error-free class as the negative class.

The ROC curves for our method, S-CNN and conventional γ-analysis are shown in Fig. 4. To generate ROC curves, for each error type, the two-class classification based on various thresholds was performed with the error-free case as the negative class and the error case as the positive class. AUC values calculated from the ROC curves are summarized in Table 1. Further, for each error type, accuracy, sensitivity and specificity obtained from the results of the two-class classification based on the ideal threshold determined from the ROC curve are shown in Table 2. In most error types other than the radiation output error, the performance of two-class classification at the ideal threshold was superior in the order of S-CNN, VAE-based method and γ-analysis.

**Table 1:** AUC of our method, S-CNN and conventional γ-analysis. AUC was calculated from ROC curves in Fig. 4; the γ-pass rate calculation was conducted with the threshold criteria of global 2%/1 mm or 3%/2 mm (DD/DTA)

	VAE	S-CNN		γ 2%/1 mm		γ 3%/2 mm	
Error type	AUC	AUC	*P*-value (vs VAE)	AUC	*P*-value (vs VAE)	AUC	*P*-value (vs VAE)
MLC (systematic)	0.952	0.999	0.028	0.953	0.867	0.921	0.327
MLC (random)	0.699	0.921	0.002	0.669	0.625	0.675	0.768
Gantry rotation (CW)	0.695	0.924	0.002	0.654	0.451	0.545	0.034
Gantry rotation (CCW)	0.741	0.928	0.006	0.725	0.740	0.607	0.041
Radiation output (+)	0.888	0.994	0.006	1.000	0.003	0.998	0.003
Radiation output (−)	0.838	0.990	0.001	0.955	0.005	0.875	0.285
Setup (lateral)	0.765	0.960	0.002	0.685	0.196	0.613	0.035
Setup (longitudinal)	1.000	1.000		0.989	0.174	0.739	<0.001
Setup (vertical)	0.901	0.983	0.031	0.830	0.081	0.549	<0.001

**Table 2:** Accuracies, sensitivities and specificities of the two-class classification performed by the proposed VAE-based, S-CNN and conventional γ-analysis methods; the two-class classification was performed for each error type, with the ‘any-error’ and error-free cases as the positive and negative classes, respectively; the ideal threshold determined from the ROC curve was used for classification in each error type; the γ-analysis was performed with the threshold criteria of global 2%/1 and 3%/2 mm (DD/DTA).

	Accuracy				Sensitivity				Specificity			
Error type	VAE	S-CNN	γ 2%/1 mm	γ 3%/2 mm	VAE	S-CNN	γ 2%/1 mm	γ 3%/2 mm	VAE	S-CNN	γ 2%/1 mm	γ 3%/2 mm
MLC (systematic)	0.889	0.986	0.903	0.861	0.917	1.000	0.861	0.889	0.861	0.972	0.944	0.833
MLC (random)	0.694	0.847	0.639	0.667	0.694	0.917	0.583	0.722	0.694	0.778	0.694	0.611
Gantry rotation (CCW)	0.694	0.875	0.639	0.556	0.694	0.972	0.611	0.583	0.694	0.778	0.667	0.528
Gantry rotation (CW)	0.736	0.861	0.708	0.597	0.778	0.944	0.750	0.556	0.694	0.778	0.667	0.639
Radiation output (+)	0.806	0.972	1.000	0.986	0.778	1.000	1.000	1.000	0.833	0.944	1.000	0.972
Radiation output (−)	0.764	0.958	0.889	0.778	0.778	0.944	0.889	0.750	0.750	0.972	0.889	0.806
Setup (lateral)	0.694	0.875	0.653	0.625	0.722	0.889	0.639	0.722	0.667	0.861	0.667	0.528
Setup (longitudinal)	1.000	1.000	0.958	0.722	1.000	1.000	0.972	0.861	1.000	1.000	0.944	0.583
Setup (vertical)	0.861	0.931	0.792	0.556	0.861	0.944	0.778	0.528	0.861	0.917	0.806	0.583

## DISCUSSION

Herein, we developed a VAE-based method for detecting errors from dose distribution and evaluated its effectiveness by performing simulations in the patient-specific VMAT QA. For most error patterns other than radiation output errors, the AUCs, accuracies and sensitivities showed that the classification accuracy was superior in the order of S-CNN ˃ VAE-based approach ˃ γ-analysis. The results of the machine learning approach, including the VAE approach, tended to outperform the conventional γ-analysis in error detection performance, a finding that was consistent with the results of previous studies. VAE-based method may be an effective solution to the problem that the models proposed in previous studies had the requirement to prepare error data in the training process. To the best of our knowledge, this is the first study that demonstrated that a machine learning approach could detect errors in dose maps without preparing the error data in advance.

Nyflot *et al.* [11] proposed a method combining CNN-based feature extraction from gamma maps and machine learning to detect MLC positional errors in patient-specific IMRT QA with EPID. Similar to our study, the authors performed simulations using systematic and random 2-mm MLC positional error plans and found sensitivities of 0.72 and 0.59 to those errors, respectively. By contrast, the sensitivities to those errors derived from our proposed method were higher (systematic error: 0.89 and random error: 0.69), which could be attributed to the different types of maps analyzed. A previous study analyzed the DD maps containing the same MLC errors by CNN in VMAT QA using Delta4 [13]. The sensitivities to systematic and random MLC position errors were 0.81 and 0.94, suggesting that our proposed method demonstrated higher sensitivity for systematic errors but lower sensitivity for random errors. In addition, that study simulated errors of similar magnitude for gantry rotation, radiation output and phantom setup. With the exception of some types of errors, results showed that supervised learning CNN tended to have higher error sensitivity than unsupervised learning-based anomaly detection methods, which also consistent with the findings of the current study, possibly indicating insufficient data. In the case of supervised learning, it was easy to learn their differences since both error-free and error images were used. By contrast, unsupervised learning-based anomaly detection quantifies anomalies in the test data based only on error-free data, which may require more training data.

As input to the VAE model, we adopted the DD map generated from the calculated and measured dose distributions. The error-free map was almost a plain image with only random error components left due to the limitations of the TPS dose calculation accuracy. Since error-free maps were used for model training, the input data with greater deviation from the plain image might have been perceived by the model as more anomalous. In fact, our method was more sensitive to phantom setup errors and systematic MLC positional errors than gantry rotation errors, which might be because the map with gantry rotation error was more similar to the error-free map than the map with phantom setup or systematic MLC positional error. The results indicate that VAE quantified how much the test map deviates from the plain image as an anomaly in the map.

In the case that the error map was input to VAE, the reconstructed map deviated significantly from the input map. Similar to the general autoencoder model, VAE reconstructs the image used for training but does not reconstruct the type of image that was not used during training. Because the VAE model was trained only on error-free data, error features were removed from the reconstructed images. In the case that the error-free map was input to VAE, it was difficult for VAE to completely reproduce the error-free map with random components in each input map, but it reproduced the average error-free map. This result was obtained because we only used error-free data to train VAE. However, the model may have responded differently had we also applied error and error-free data in the training process. In that case, it may be possible to perform error classification based on the distribution of latent variables since feature extraction is conducted to reproduce the features of the input map for each pattern on the reconstruction map. We plan to investigate this in the future.

Although we constructed a typical VAE model for this study, the architecture of the VAE model may be further improved to achieve even better classification accuracy. While it is to be expected that changes in network architecture and complexity will affect classification performance, changing the number of latent dimensions is a particularly interesting attempt. Herein, the two-class classification was performed based on the 30D feature vectors obtained from the encoder; however, it was unclear whether the 30 dimensions adopted here were the best setting. Generally, in the case wherein the latent dimension is extremely small, the information about the input data cannot be completely compressed, although a single dimension can hold a lot of information. Conversely, when the latent dimension increases, the latent variable sufficiently retains the information of the input data, but the amount of information per dimension decreases. Therefore, searching for the best architecture should be considered as a future step toward improving the performance of the model. In addition, there is room to consider the input image size. In this study, 5-mm resolution images were used as the input to avoid inaccurate information being reflected in the map due to interpolation processes. However, input images of higher resolution would enable more information to be retained, which may improve the model’s performance. Although it is necessary to devise an interpolation method to eliminate inaccurate information as much as possible, changing the size of the input image is an interesting approach.

This study has several limitations that should be addressed in the future. First, the analysis target plan was limited to the prostate VMAT plan. Prostate VMAT is a widely used basic irradiation technique with a relatively simple dose distribution. Since this study is the first report which applies the unsupervised deep learning-based anomaly detection method to the dose-distribution analysis, we limited our analysis to the prostate VMAT plan to evaluate the basic performance of our method. However, testing the method on VMAT plans for a variety of irradiated sites would be an important next step to assess the versatility of the proposed method. Second, simulated error patterns in this study were limited and did not cover the variation of errors that could actually occur in practice. For example, rotation errors were simulated only in the gantry rotation direction, but phantom setup errors in the pitch and yaw directions may occur in clinical practice. For MLC error simulation, a 2-mm offset was applied to all leaves. However, realistic leaf positional errors are expected to be to be in the sub-millimeter order and are more likely to occur on a single leaf, as opposed to all leaves. In addition, the magnitude of errors that realistically occur is not constant, and two or more errors may occur at the same time. Consequently, simulations with such error patterns would allow a more accurate evaluation of the applicability of this method. Third, the performance of our method may be affected by intrinsic uncertainties that exist in the treatment system. The training data included error components due to the limits of accuracy of the dose calculation algorithm and beam modeling as well as small mechanical error components that were not completely zero. The clinical uncertainties vary from facility to facility. Therefore, it may be possible to evaluate the performance of our proposed method in more detail by performing simulations using linac and TPS from other facilities. This future work is an essential step toward implementing the clinical usage of this model.

## CONCLUSION

A VAE-based method to detect error from the analysis of DD maps was constructed, and its performance was evaluated via simulations in the patient-specific VMAT QA. This model could be trained only with error-free data and could detect errors in the dose map by quantifying the anomaly of an input map. Our results indicated that our method is an effective solution to this shortcoming in the patient-specific VMAT QA.

## CONFLICT OF INTEREST

There is no conflict of interest with regard to this manuscript.

## FUNDING

This work was supported by the Foundation for Promotion of Cancer Research in Japan.
